# COVID-19 morbidity in lower versus higher income populations underscores the need to restore lost biodiversity of eukaryotic symbionts

**DOI:** 10.1016/j.isci.2023.106167

**Published:** 2023-02-09

**Authors:** William Parker, Esha Patel, Kateřina Jirků-Pomajbíková, Jon D. Laman

**Affiliations:** 1 WPLab, Inc., Durham, NC, USA; 2 Institute of Parasitology, Biology Centre, Czech Academy of Sciences, 370 05 České Budějovice, Czech Republic; 3 Faculty of Science, University of South Bohemia, 370 05 České Budějovice, Czech Republic; 4 Department of Pathology and Medical Biology, University Groningen, University Medical Center Groningen, Groningen, the Netherlands

**Keywords:** Health sciences, Biological sciences, Virology

## Abstract

The avoidance of infectious disease by widespread use of ‘systems hygiene’, defined by hygiene-enhancing technology such as sewage systems, water treatment facilities, and secure food storage containers, has led to a dramatic decrease in symbiotic helminths and protists in high-income human populations. Over a half-century of research has revealed that this ‘biota alteration’ leads to altered immune function and a propensity for chronic inflammatory diseases, including allergic, autoimmune and neuropsychiatric disorders. A recent Ethiopian study (EClinicalMedicine 39: 101054), validating predictions made by several laboratories, found that symbiotic helminths and protists were associated with a reduced risk of severe COVID-19 (adjusted odds ratio = 0.35; p<0.0001). Thus, it is now apparent that ‘biome reconstitution’, defined as the artificial re-introduction of benign, symbiotic helminths or protists into the ecosystem of the human body, is important not only for alleviation of chronic immune disease, but likely also for pandemic preparedness.

## Graphical abstract

**Figure:**
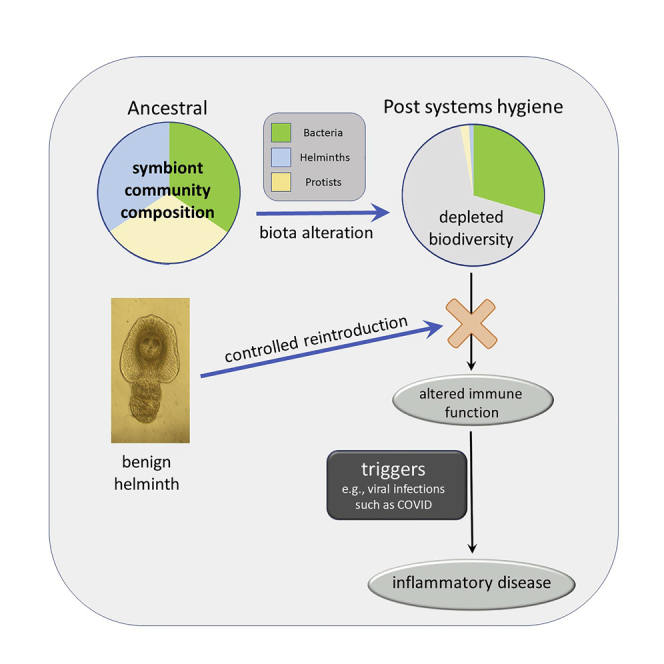

## Introduction

This narrative review discusses the intersection between biodiversity in the human body and the COVID-19 pandemic. Factors in areas of the world with high average income that affect biodiversity in the human body have been discussed previously in detail.^1^^,^^2^^,^^3^ In that discussion, a useful distinction can be made between different types of hygiene and their effects on biodiversity.^3^ Personal hygiene is important for preventing transmission of acute infections such as common colds, SARS-CoV-2 and many other communicable diseases that human populations have experienced following the agricultural revolution and subsequent urbanization.^1^ These infectious agents generally colonize humans only transiently, and generally induce inflammatory responses in their hosts. In contrast, “systems hygiene” has an independent and distinct effect on biodiversity.^3^ Systems hygiene, which includes the use of sewage systems, water treatment facilities, and food processing and storage technologies, effectively deplete particular components of the biome, most notably helminths. In contrast to bacterial and viral pathogens, helminths generally colonize humans for extended periods of time and have a regulatory effect on immune function.^1^

The impact of helminths on immunoregulation has been the subject of considerable study for decades.^2^^,^^4^^,^^5^^,^^6^^,^^7^^,^^8^ Underlying mechanisms are multifactorial, and include three general types of effects. First, helminths secrete and excrete dozens if not hundreds of immunoregulatory molecules that directly affect host immune function. Second, helminths stimulate the production of negative feedback or regulatory systems that would otherwise remain unstimulated. Finally, helminths stimulate and activate immune components such as eosinophils and IgE-producing B cells that would otherwise remain unstimulated. This “compartment utilization” may help avoid deployment of those components in pathologic responses involving autoimmune and allergic reactions.

In a recent discussion,^3^ we examined the intersection between viral infections, which are decreased by personal hygiene, and exposure to helminths, which can be eliminated by widespread use of systems hygiene. The view that the presence of helminths attenuates hyperinflammatory and autoimmune responses triggered by viral infections has a long history and is well supported in the literature.^3^ Although we and others hypothesized that helminths would attenuate hyperinflammatory and detrimental responses to the SARS-CoV-2 virus,^3^^,^^9^^,^^10^^,^^11^^,^^12^^,^^13^^,^^14^^,^^15^^,^^16^ the discussion was hampered by a lack of clarity regarding the impact of biodiversity of the COVID-19 pandemic.^17^^,^^18^ With that in mind, this narrative review will place particular emphasis on the results of a recent Ethiopian study that addressed the impact of biodiversity within the human body on clinical outcomes of SARS-CoV-2 infection.^19^ That study and the implications of that study in the context of previously published literature will be discussed.

## The intersection of biodiversity and COVID

During the first 12 months after the emergence of the SARS-CoV-2 virus and the resulting COVID-19 pandemic, numerous predictions were made regarding the potential effects of the virus on areas of the world with low average income and relatively little infrastructure for medicine and sanitation. Based on the impact of the pandemic in areas with high-income, particularly the US and parts of Europe, it was generally recognized that the younger average age of individuals in low-income areas would be protective.^20^ However, this advantage was expected to be offset by disparities in medical care, resources for personal hygiene, infrastructure-based capacity to shut down day-to-day activity, and co-occurrence of AIDS and other infectious disease. Thus, the clinical impact of the virus was expected by many to be much greater in low-income areas of the world than in high-income areas.^20^^,^^21^^,^^22^^,^^23^^,^^24^^,^^25^^,^^26^^,^^27^ On the other hand, it was also recognized that, in high income areas, severe reactions to the SARS-CoV-2 virus were generally underpinned by hyper-responsiveness of immune function with an autoimmune-like signature (reviewed by Halpert and Shoenfeld^28^).

**Figure 1:**
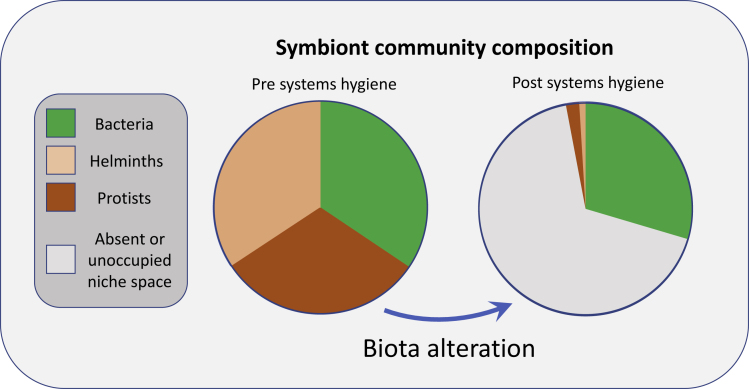
Biota alteration caused by systems hygiene This diagram shows relative changes in community composition of major clades of symbionts as a function of systems hygiene. The diagram does not include clades, including viruses and yeast, for which little information is available regarding the impact of post-industrial lifestyle in general and systems hygiene in particular. The three clades are arbitrarily given equal weight in the ancestral state, and that weight does not reflect the number of species in a clade, the space occupied by the clade within the ecosystem, the biomass of the clade within the ecosystem, or the importance of the clade for host metabolism or immune system development. The niche space left by depletion of the vast majority of helminths and protists is unoccupied. Loss of microbial diversity is relatively minor by comparison,^38^ and is because of loss of niche space as a consequence of decreases in consumption of dietary fiber, the primary food source for much of the symbiotic bacteria.^38^^,^^39^^,^^40^^,^^41^

**Figure 2:**
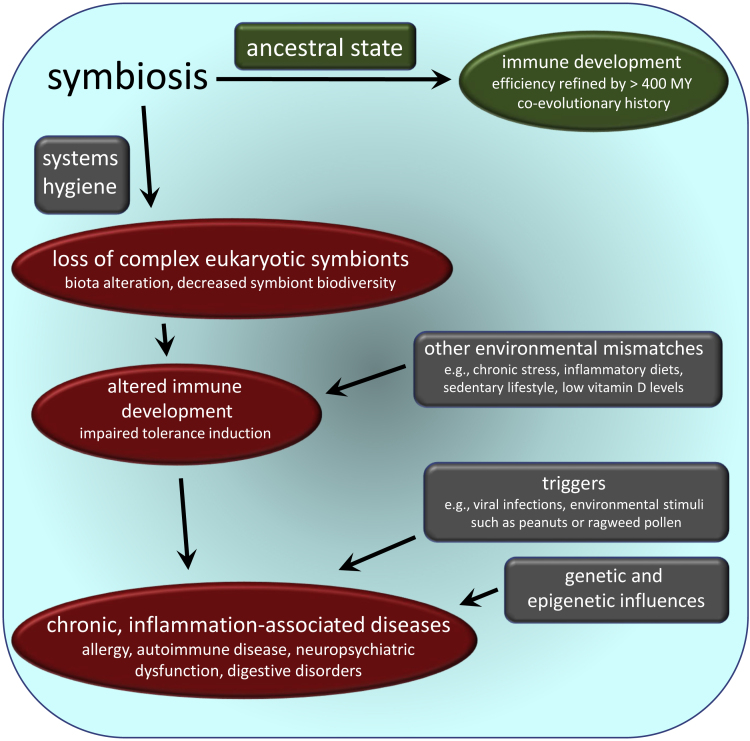
Biota alteration and its connection with chronic, inflammation-associated diseases In this model, loss of complex eukaryotic symbionts leads to altered immune regulation, particularly impaired ability to achieve tolerance to self and environmental antigens. This condition, when combined with (1) other environmental mismatches, (2) “triggers” such as viral infections, and (3) genetic and epigenetic factors, leads to chronic inflammation-associated disease. In this case, “systems hygiene” is defined as factors such as sewage treatment facilities, clean water utilities, and food storage technology that effectively prevent water-borne disease.^3^ Although systems hygiene causes biota alteration and subsequent risks for immune dysregulation, personal hygiene can decrease exposure to some triggers for chronic disease, including insect-derived antigens and viral infections.^3^^,^^34^^,^^35^^,^^36^

The hyper-responsive, autoimmune-like nature of severe COVID-19 disease provided a critical clue to the potential impact of the virus in low-income areas. Data emerged more than half a century ago pinpointing “biota alteration”, changes in the community of symbiotic life associated with the human body, as the critical element in the emergence of chronic inflammatory disease in high-income countries.^29^^,^^30^^,^^31^^,^^32^^,^^33^ In particular, a profound depletion of symbiotic helminths and protists (Figure 1) has been identified as critical in the high prevalence of chronic, inflammation-associated diseases in high-income areas^29^^,^^30^^,^^31^^,^^32^^,^^33^^,^^34^^,^^35^^,^^36^ (Figure 2). In this context, helminths and protists can be considered together in a group as “complex eukaryotic symbionts”, despite the fact that the group includes all three major classes of symbiotic helminths, trematodes (flukes), cestodes (flatworms) and nematodes (roundworms) as well as an incredibly diverse array of protists.^37^ For a list of definitions, see Box 1.

Box 1 **Definitions**

• Symbiont: An organism living in close physical association with another organism. Symbiotic relationships may be parasitic or mutualistic in nature, and the nature of a particular symbiotic relationship may be conditional, depending on environmental conditions and other factors.^29^^,^^34^
• Host: A symbiont that is typically larger and provides habitat to another, typically smaller, symbiont.
• Parasite: A symbiont that derives benefits from its host while harming its host.
• Infection: A symbiotic relationship in which a symbiont, usually a parasite, causes a disease or pathological state in the host as a result of living on or within the host.
• Mutualist: A symbiont that derives benefits from its host while also providing benefits to its host.
• Systems hygiene: Community-wide use of technologies that prevent transmission of pathogens. This type of hygiene involves water purification, sewage and waste management, food production and storage, modern building construction methods, and other technologies. It is distinguished from personal hygiene measures such as social distancing, wearing of face coverings, and reducing hand-to-face contact.
• Helminth: The adult stage of a fluke (trematode), flatworm (cestode) or roundworm (nematode) living as an obligate symbiont in a vertebrate.
• Complex eukaryotic symbionts: Symbiotic helminths and protists. This group does not include fungi and yeast, and it is typically depleted by systems hygiene.
• Biota alteration: Changes imposed by systems hygiene on the community composition of symbionts associated with the human body. The primary change of medical importance associated with biota alteration is the virtually complete loss of complex eukaryotic symbionts.
• Biota reconstitution: The intentional, systematic, and controlled introduction of complex eukaryotic symbionts to a population which has been largely depleted of those symbionts.
• Biota maintenance: The intentional, systematic maintenance of complex eukaryotic symbionts in a population which already has complex eukaryotic symbionts, either through natural transmission or through biota reconstitution.
• Environmental mismatch: An environmental change that leads to disease. Classic examples include the availability of processed foods and labor-saving technology. These mismatches lead to consumption of inflammatory diets and sedentary lifestyles, respectively, which in turn cause disease. Another example is the widespread use of systems hygiene, which in turn leads to biota alteration and subsequent disease.

Complex eukaryotic symbionts, despite being diverse in nature, share some features in common that are relevant for this discussion. Complex eukaryotic symbionts tend to be larger in size than bacteria and yeast, although exceptions do exist. These larger symbionts are poorly or ineffectively phagocytized by the host immune system, often eliciting phagocytosis-independent immune responses in their hosts. These immune responses include IgE secretion,^42^ mast cell activation,^43^ eosinophil production,^44^ and neutrophil extracellular trap (NET) deployment,^45^^,^^46^ and are generally distinct from those elicited by symbiotic bacteria and yeast. At the same time, the nature and intensity of the host response to complex eukaryotic symbionts generally depends on the habitat of the symbiont, with symbionts in the gut eliciting a diminished response compared to extra-intestinal symbionts.

Another factor that all complex eukaryotic symbionts have in common is that they are extensively depleted from the human biome by widespread use of “systems hygiene”.^3^ Systems hygiene includes factors such as water purification plants and sewage treatment systems that prevent oral-fecal transmission of complex eukaryotic symbionts between hosts. In addition, systems hygiene includes food treatment and storage technologies that prevent access to food supplies by insects, rodents and other species that might effectively transmit complex eukaryotic symbionts. In contrast, symbiosis with bacteria and yeast is largely independent of systems hygiene, and is rather determined for the most part by parent-to-offspring transmission^47^ and host diet.^38^^,^^48^^,^^49^^,^^50^

Although systems hygiene is absolutely necessary to avoid transmission of deadly infectious diseases, the accompanying biota alteration characterized by a dramatic decrease in complex eukaryotic symbionts has substantial implications for public health. Pandemics of allergic disorders, autoimmune disease, and digestive disorders result.^34^^,^^36^^,^^51^^,^^52^^,^^53^^,^^54^^,^^55^ In addition, neuropsychiatric conditions such as major depressive disorder and anxiety disorders are apparently a result, at least in part, from systems hygiene-associated biota alteration.^56^^,^^57^^,^^58^^,^^59^^,^^60^

## The impact of biota alteration on COVID-19

The impact of complex eukaryotic symbionts on the immunological response to COVID-19 in humans has not yet been evaluated, with studies to-date being limited to clinical outcomes. However, Shoenfeld et al.^61^ noted that severe cases of COVID-19 and autoimmune diseases share pathologic mechanisms and some aspects of radiological assessment in the clinic. Uncontrolled immune activation with excessive cytokine release was described in severe cases of COVID-19, with a number of proposed mechanisms such as epitope spreading and presentation of cryptic antigens that are hallmarks of autoimmune disease.^61^ Later *in-vitro* studies using T cells from COVID-19 patients showed that helminth derived products significantly reduced the frequency of SARS-CoV-2-reactive CD4^+^T cells, whereas the frequency of SARS-CoV-2-reactive CD8^+^T cells was unaffected or even increased.^62^ In addition, stimulation *in vitro* with helminth derived products led to an increase in IL-10 production and a reduction in IFNγ and TNFα, suggesting that helminths may impact the cytokine storm observed in many cases of severe COVID-19, and that the presence of helminths diminishes the helper T cell-mediated immune response to SARS-CoV-2, but has little or no effect on the magnitude of virus-specific cytotoxic T cell responses. However, as Shoenfeld et al. point out, cytotoxic T cell responses are involved in autoimmune disease and in COVID-19 responses,^61^ and, given the impact of helminths on autoimmune processes, it is not surprising that the presence of helminths has an effect on the function of CD8^+^T cells.^63^ Indeed, studies in laboratory animal models suggest that helminths can stimulate CD8^+^T cells in a manner that enhances immunity to both viral and bacterial infections.^64^^,^^65^ Thus, helminths exert a broad influence on immunity, affecting both T helper and cytotoxic T cell function that may be important in the response to SARS-CoV-2.

Given that (1) biota alteration leads to increases in chronic inflammatory conditions including autoimmune disease, and (2) morbidity and mortality are associated with hyperinflammatory, autoimmune-like responses to SARS-CoV-2 in areas with widespread use of systems hygiene, it was predicted by several groups that the presence of complex eukaryotic symbionts would attenuate the clinical impact of the SARS-CoV-2 virus. To evaluate the number of laboratories making this prediction, a systematic literature search was conducted on October 31, 2022 examining the Medline database and all manuscripts with the terms “helminth” and “COVID”. The term helminth was used to the exclusion of other complex eukaryotic symbionts because available data in humans up until 2019 regarding attenuation of chronic inflammatory processes was limited to helminths (See Table 1 and further discussion below). The review yielded a total of 108 results and revealed 8 different groups predicting that helminths would attenuate the clinical symptoms of COVID-19^3^^,^^9^^,^^10^^,^^11^^,^^12^^,^^13^^,^^14^^,^^15^^,^^16^prior to the emergence of experimental data addressing the issue in July of 2021.^19^ However, it was also expected that such attenuation might be masked by difficulties in reporting from low-income areas and by demographic factors such as age and comorbidities. Nevertheless, as the COVID-19 pandemic progressed during 2020, several studies noted a relatively light clinical impact of the pandemic in developing countries.^66^^,^^67^^,^^68^^,^^69^^,^^70^^,^^71^ In addition, a multivariate analysis of publicly available data from 106 countries^72^ found that, independent of demographic factors, the presence of improved hygiene and the higher incidence of autoimmune disease were associated with increased mortality due to COVID. However, underreporting in low-income areas of morbidity and mortality because of COVID-19 remained a concern, and immune-regulating factors other than the presence of complex eukaryotic symbionts were considered as possible explanations. For example, protection mediated by previous exposure to other coronaviruses, BCG vaccination, and genetic variation were proposed as factors that might account for the apparently low impact of SARS-CoV-2 in countries without widespread use of systems hygiene.^18^^,^^66^^,^^73^

**Table 1:** Beneficial effects of exposure to helminths in humans

Organism	Species	Beneficial effects in humans
Cestodes	*Hymenolepis nana*	- A reduced risk of severe reactivity to SARS-CoV-2 was observed in individuals with natural exposure.^19^ - The progression of relapsing remitting multiple sclerosis was halted following natural exposure.^74^^,^^75^^,^^76^ a
	*Hymenolepis diminuta*	- Effective treatment of some allergic disorders including seasonal allergies,^77^^,^^78^ depression and anxiety disorders,^56^ and some autoimmune conditions^77^^,^^78^ was reported in individuals self-treating with helminths.
Nematodes	*Schistosoma mansoni*	- A reduced risk of severe reactivity to SARS-CoV-2 was observed in individuals with natural exposure.^19^ - A reduced course of asthma was observed in individuals with natural exposure.^79^ However, in general, current exposure to helminths is not associated with protection against asthma.^80^
	*Ascaris lumbricoides*	- A reduced risk of severe reactivity to SARS-CoV-2 was observed in individuals with natural exposure.^19^ - The progression of relapsing remitting multiple sclerosis was halted following natural exposure.^74^^,^^75^^,^^76^ a - Seasonal allergies were eliminated following natural exposure, but returned after removal of the helminth.^29^
	*Trichuris trichiura*	- A reduced risk of severe reactivity to SARS-CoV-2 was observed in individuals with natural exposure.^19^ - The progression of relapsing remitting multiple sclerosis was halted following natural exposure.^74^^,^^75^^,^^76^ a - Remission of ulcerative colitis was achieved as a result of self-treatment by one patient.^81^^,^^82^
	*Strongyloides stercoralis*	- The progression of relapsing remitting multiple sclerosis was halted following natural exposure.^74^^,^^75^^,^^76^ a
	*Enterobius vermicularis*	- The progression of relapsing remitting multiple sclerosis was halted following natural exposure.^74^^,^^75^^,^^76^ a
	*Necator americanus*	- A possibly reduced risk of severe reactivity to SARS-CoV-2 was observed in individuals with natural exposure.^19^ b - Individuals with natural exposure may be protected from asthma.^80^ However, in general, current exposure to helminths is not associated with protection against asthma.^80^ - Some indications of possible improvement were seen in patients with Crohn's disease in a small clinical trial, but attempts to remove patients from immunosuppression and maintain remission from Crohn's disease were not successful.^83^ - No therapeutic effects on allergic rhinoconjunctivitis without asthma were observed following low levels of exposure in a clinical trial.^84^ - An average improvement in asthma was observed following low level exposure in a clinical trial, but results were not significant compared to controls.^85^ - Some biomarkers for celiac disease were affected in a positive way by low levels of exposure in a clinical trial, but actual clinical measures of celiac disease were not affected.^86^ - Effective treatment of some allergic and autoimmune conditions was reported in individuals self-treating with helminths.^77^^,^^87^
	*Trichuris suis*	- Remission from Crohn's disease was achieved in more than 70% of individuals in an initial, uncontrolled clinical trial.^88^ However, probably for a number of reasons (see Box 2), results have not been reproducible.^89^^,^^90^ - Statistically significant reductions in the disease index for ulcerative colitis have been achieved compared to controls in a clinical trial.^82^^,^^91^ However, larger trials were shut down prior to completion, and results have not been reported. - Some benefits in terms of progression of disease as measured by MRI and in terms of immunoregulatory markers might be achieved for some patients with multiple sclerosis in clinical trials,^92^ but results are overall modest^92^ or possibly even negative,^93^ and considerable variation exists.^92^ - Beneficial effects with a large effect size were observed in a small clinical trial when monitoring repetitive behaviors, restricted interests, and irritability of adult patients with autism spectrum disorder.^94^ However, no effects on social/communication parameters were observed, and none of the effects were statistically significant when compared to controls due to small sample size and large deviations in the data.^94^ - Effective treatment of some allergic and autoimmune conditions as well as some neuropsychiatric conditions was reported in individuals self-treating with helminths.^56^^,^^77^ However, data from self-treatment using this helminth are less extensive than data involving self-treatment with either *Hymenolepis diminuta* or *Necator americanus*.

A reduced risk of severe reactivity to SARS-CoV-2 was also observed in individuals with natural exposure to various protists, including *Entamoeba histolytica*^19^, *Giardia intetinalis*^19^, *Plasmodium* species^95^^,^^96^, and *Leishmania* species^97^. Most of the symbionts shown can be pathogenic (disease inducing) in humans. The exceptions are *Hymenolepis diminuta* and probably *Trichuris suis*, neither of which are naturally occurring symbionts in humans and both of which have been artificially introduced into humans for the purpose of helminth therapy.^98^ A given symbiont can have multiple effects, and various symbionts can have the same effect. Although the protective effect of protists has not been well established in humans, the issue has been addressed in laboratory animal models (see text). The type of exposure, whether (a) natural (unintentional, accidental) acquisition, (b) exposure during a clinical trial, or (c) self-treatment (intentional self-exposure with the intent to treat disease) is indicated. In general, natural exposures and self-treatments are more successful than are clinical trials, probably for several reasons (see Box 2). Most of the symbionts shown are pathogenic (disease inducing) in humans.

a The progression of relapsing remitting multiple sclerosis was “halted” as defined by elimination of increases in disability status over a period of 5 years^75^ and no significant changes in either the number of new or enlarging T2 lesions or the number of Gd-enhancing lesions over a period of 7.5 years.^76^

b The species of hookworm was not described in the study, and the trend was not statistically significant.^19^

## Experimental evidence of the protective effect of complex eukaryotic symbionts on the clinical progression of COVID

In mid-2021, a study conducted in Ethiopia addressed the predictions made that complex eukaryotic symbionts would protect individuals from severe COVID-19.^19^ In that study, Dewit Wolday, Tobias Rinke deWit and 19 Ethiopian colleagues evaluated the impact of SARS-CoV-2 on 751 Ethiopian patients, 284 of whom had complex eukaryotic symbionts (helminths and/or protists) living in their intestines. After correction for a variety of factors including age, education level, and comorbidities, Wolday et al. found that the presence of complex eukaryotic symbionts was associated with an odds ratio of 0.35 (0.26–0.48; p<0.0001) for severe COVID-19.^19^ They also found that, although no patients with complex eukaryotic symbionts died, 2.4% of patients without complex eukaryotic symbionts died from COVID-19.^19^

Because the Wolday et al. study^19^ was the first and still is the only study to directly examine the impact of helminth colonization on the clinical progression of COVID, the study merits careful examination. It was cited 20 times based on a Medline query made October 31, 2022, with 17 of those studies considering the impact of complex eukaryotic symbionts on COVID-19. Of those 17, a large majority of 14, including one written by Wolday, accepted that Wolday had established an association between helminth colonization and better clinical outcomes following SARS-CoV-2 infection. One opinion piece referred to Wolday’s study as “preliminary”,^99^ although it was not clear why the study could be considered preliminary. Indeed, a completed data collection and analysis with exceptional statistical significance were described in the Wolday report. Two groups questioned the selection of patients in the Wolday study,^100^^,^^101^ both objecting to the conclusion that helminths could be protective of COVID. In one case, the cohort evaluated by Wolday was said to be non-representative of the whole population.^100^ In the other case, the sample collection method was said to be “biased” in an unspecified manner.^101^

Wolday’s methods^19^ involved recruitment of individuals with positive COVID-19 tests from a variety of sources: For example, from patients admitted to the hospital, from mass screening of travelers and people who had come into contact with COVID-positive individuals, and from screening of health care workers. Although a cohort selected in this manner may not be representative of the population as a whole, any difference between the cohort studied and the Ethiopian population in general does not invalidate the results obtained by Wolday. Indeed, given the methods used, it is difficult to envision any selection bias that would invalidate the study. It could be hypothesized that patients who had severe reactions to helminths had already had their helminths removed, and these patients, whose immune systems were somehow different as indicated by their adverse reaction to helminths, were more susceptible to COVID. However, this possibility seems unlikely given a survey of the Ethiopian population revealing that fewer than 1% of individuals are treated in clinics for helminth infections each year,^102^ and findings that helminths persist in Ethiopia despite mass deworming campaigns with anti-helminthic drugs.^103^^,^^104^ Furthermore, patients in Wolday’s study without helminths had *more* gastrointestinal symptoms than patients with helminths, consistent with the view that low levels of helminth colonization are often asymptomatic and thus do not necessitate medical intervention. Finally, multivariate analysis was used to control for numerous factors including age, sex, comorbidities and residence (potentially correlated with social status) that likely affect immune function and/or helminth colonization,^105^^,^^106^^,^^107^^,^^108^ reducing the possibility that a confounding factor or factors could account for the dramatic results obtained.

Given a lack of alternative explanations for Wolday’s data, and given the prediction of the results by 8 independent research groups, we argue that Wolday’s results can be taken at face value: the presence of helminths is associated with less severe clinical outcomes in COVID. Nevertheless, further studies in other human populations would certainly be worthwhile to determine how broadly applicable Wolday’s observations are. At the same time, the view that helminths attenuate hyperinflammatory responses as seen in severe COVID-19 has a firm scientific basis dating back more than 50 years,^3^ and *in vitro* studies have recently confirmed at least some aspects of the mechanisms underlying attenuation of the anti-COVID-19 response in particular.^62^ Thus, we conclude that, in light of Wolday’s study,^19^ predictions made by multiple laboratories are correct, and helminths do indeed attenuate the hyperinflammatory response induced in some individuals by the SARS-CoV-2 virus, thereby reducing the clinical impact of the virus.

## A remarkable effect of biota alteration on the pathogenesis of COVID, but do we know the full extent of it?

The fact that Wolday noted an adjusted odds ratio (aOR) of 0.35 (0.26–0.48; p<0.0001) for severe COVID-19 in the presence of complex eukaryotic symbionts in Ethiopia^19^ is remarkable, in light of the fact that their study evaluated only active colonization with intestinal symbionts as measured by microscopy of fecal samples. Almost 38% of their patients had some active colonization with at least one complex eukaryotic symbiont, and, with such high colonization rates, it might be supposed that many of the other patients, even if they did not presently have active colonization, would have been exposed in the past and would still retain some level of immune modulation based on that past exposure. Furthermore, as pointed out to us by the senior author of the paper, Tobias Rinke deWit, more helminths and protists would likely have been found if the authors had employed molecular technologies rather than microscopy to assess their presence. Indeed, current data indicate that the blood-borne protist responsible for malaria can alter immune function in a way that may be protective against SARS-CoV-2 infection.^95^^,^^109^ This view brings up the possibility that the level of protection may have been much higher (that the 0.35 odds ratio may have been much smaller) if Wolday had been able to compare individuals colonized with complex eukaryotic symbionts compared to typical individuals living in high income countries who had never been exposed to such symbionts. A caveat to this hypothesis is that no biomarkers for past exposure to complex eukaryotic symbionts were assessed in Wolday’s study, and thus it remains unknown to what extent the non-colonized patients in the study had previously been exposed to complex eukaryotic symbionts. However, exposure seems likely in light of the high colonization rate of the patient population.

## Additional impetus for biota reconstitution and maintenance

The results found by Wolday et al.^19^ add further credence to the view that complex eukaryotic symbionts should be artificially reintroduced into populations with widespread use of systems hygiene. This approach of “biome reconstitution”, now more than a decade old,^110^ involves the domestication and cultivation of benign eukaryotic organisms in much the same way as some bacteria are maintained and cultivated by the probiotic industry. For such reconstitution, helminths are considered superior to protists because tight control of the reproduction and dissemination is often feasible in a typical high-income country. In addition to the feasibility of controlling their reproduction and dissemination, various selection factors for candidate helminths have been considered.^98^ Furthermore, the benefit to cost ratio of a given helminth can be conditional^34^: An individual who is malnourished and has multiple parasitic species will likely not benefit from any helminth, whereas an individual with autoimmune and neuropsychiatric conditions who is raised in an environment with extensive systems hygiene may well receive significant benefits from particular helminths while experiencing negligible adverse effects.^57^^,^^74^^,^^78^^,^^111^ In those cases where the benefits outweigh the costs, helminths, by definition, are classified as therapeutic rather than parasitic or pathogenic.

Such reconstitution is expected to alleviate a wide range of allergic, autoimmune, and digestive conditions. In addition, biome reconstitution may help prevent or even treat some neuropsychiatric conditions such as depression and anxiety disorders.^56^^,^^57^^,^^58^^,^^77^^,^^78^^,^^111^ Wolday’s study adds to the list of potential benefits of such reconstitution, pointing toward the potential of human populations to be more resistant to pandemics of viral disease.^3^ In this view, symbiotic relationships can be situational, with an organism being a parasite in one context, and a mutualist in another.^34^^,^^112^ Although some idea of how such reconstitution can be conducted is evident, many knowns and unknowns remain. For example, it is expected that helminths rather than protists may be the most useful for initial clinical trials because the growth, reproduction, and transmission of helminths is easier to control than that of protists. However, it remains unknown which helminths will have the best cost/benefit ratios, and what factors might affect the cost/benefit ratio for a particular helminth in a particular patient (Box 2: Knows and unknowns of biome reconstitution).

Box 2 **Knows and unknowns of biome reconstitution**

Knowns
  • It should work. Several studies^29^^,^^74^^,^^75^^,^^76^ have shown that some disease states associated with biota alteration are reversed by natural acquisition of complex eukaryotic symbionts, particularly helminths.
  • It can work. Evaluation of the effects of intentional self-treatment with helminths shows that some disease states associated with biota alteration can be reversed by intentional biome reconstitution.^56^^,^^77^^,^^78^
  • It has not worked yet, at least not very well. Clinical trials of biome reconstitution have shown lackluster results, and biome reconstitution has not yet reached the mainstream. The likely reasons appear to be inadequate preparation of the symbiotic organisms for clinical trials and a one-dose-fits-all approach to clinical trials that is ineffective for biome reconstitution.^111^
Unknowns
  • How well does it work? Although prior work with accidental exposure to symbionts and self-treatment with symbionts has proven informative, certainty regarding the effectiveness of biota reconstitution for various diseases will require evaluation through well designed clinical trials which have yet to be conducted.
  • Which organisms work best? The selection of complex eukaryotic symbionts for previous attempts at biome reconstitution has been haphazard at best, and only a few organisms have been evaluated.^98^ It remains unknown which species will have better benefit to cost ratios, and whether particular species or combinations of species will work better for particular diseases.
  • How can it be done? Although the first published evidence pointing to the importance of biome reconstitution for human health is now more than 50 years old, the pathway to biome reconstitution is still impeded by financial hurdles,^113^^,^^114^ difficulties in symbiont preparation,^111^ and ineffective clinical trial designs.^111^ However, it is anticipated that significant investment from either commercial interests or not-for-profit benevolent organizations would be significant to accomplish the goal.

## Implications for biome alteration and reconstitution: Continuous exposure is probably needed to maintain function

Wolday’s study^19^ has several implications for ongoing efforts to restore the human biome with the goal of preventing chronic inflammatory diseases or mitigating the impact of future viral pandemics (pandemic preparedness). The fact that Ethiopian patients not presently colonized with a complex eukaryotic symbiont were more susceptible to severe COVID-19 is consistent with the view that continuous exposure to such symbionts is beneficial. Here again the caveat applies to the Wolday study: prior exposure of non-colonized individuals to complex eukaryotic symbionts in Ethiopia seems likely, but it was not assessed in the study. However, chronic inflammatory diseases increase in immigrants moving from a low-income to a high-income country,^115^^,^^116^ supporting the view that consistent exposure to complex eukaryotic symbionts is necessary for healthy immune function.

This conclusion is further supported by Zvi Bentwich’s work with Falasha Jews who migrated from Ethiopia to Israel in several waves in the 1980’s and 1990’s.^117^ Bentwich’s team found that, six months following removal of helminths, the immune system of immigrants from Ethiopia to Israel changed from a low-sensitive system characteristic of new immigrants to a high-sensitive system characteristic of long-term residents in Israel. Furthermore, Ethiopian immigrants who had lived in Israel for 3–10 years had immune systems that were more sensitive than immigrants who had been in Israel for 1 year or less.^117^ These data again support the view that efforts at biome reconstitution should entail continued exposure for extended periods of time if not the life of the individual. Such “biome maintenance” is expected to be particularly important in the prevention of chronic inflammatory disease, an approach that is preferable to reactive, treatment-oriented medical approaches.

## Implications for biome alteration and reconstitution: The absolute presence or absence of complex eukaryotic symbionts rather than the presence of particular species may be critical for immune function

Woldays’ study^19^ has implications for other aspects of biome reconstitution. Wolday et al. showed that all three classes of complex eukaryotic symbionts (flatworms, roundworms, and protists) were independently protective from severe SARS-CoV-2. This finding might suggest that any complex eukaryotic symbiont “fills the gap” in immune system training and restores the biome for clinical intents and purposes. In support of this view, exposure to protists from the *Plasmodium* group, the blood-borne pathogens that are the causative agents for malaria, were not monitored in Wolday’s study, but several studies suggest that these organisms are protective against COVID. For example, healthcare workers with exposure to *Plasmodium* species cleared the SARS-CoV-2 virus faster than uninfected individuals, with mean clearance times of 8 days and 12 days, respectively (p < 0.005).^95^ In addition, high levels of exposure to *Plasmodium falciparum* were associated with less severe/critical COVID-19 and fewer adverse outcomes.^96^ Similarly, prior exposure to the protist causing leishmaniasis, again not monitored in the Wolday study, was associated with a very high degree of protection (OR =0.12, CI: 0.03–0.30 and p<0.001) from COVID-induced morbidity.^97^

The view that the presence of any complex eukaryotic symbiont rather than one particular symbiont is important for immune function is not new. For example, the study of multiple sclerosis (MS) in Argentinian patients with and without complex eukaryotic symbionts strongly supported that view.^74^^,^^75^^,^^76^ In that study, one species of cestode (a flatworm) and four species of nematodes (roundworms) effectively halted the progression of relapsing remitting MS in patients, completely eliminating increases in disability status over a period of five years that were seen in controls without helminths.^75^ In their 7.5-year follow-up study, investigators noted that MS had begun to progress in patients who had eliminated their helminths using anti-helminthic therapy, but no significant changes in either the number of new or enlarging T2 lesions or the number of Gd-enhancing lesions were observed in patients who still had helminths.^76^ In another example, the roundworm symbiont *Ascaris lumbricoides* was shown to alleviate seasonal allergies more than 50 years ago,^29^ and dozens of individuals are now using the benign (non-pathogenic) flatworm *Hymenolepis diminuta* to alleviate seasonal allergies.^77^^,^^78^

The Wolday study^19^ adds to a growing body of evidence suggesting that a wide range of complex eukaryotic symbionts (flatworms, roundworms, and protists) exert similar effects on the mammalian immune system. These phylogenetically distant symbionts may fill the same “niche space for immune function” because of selection pressures forcing the organisms to adapt to immune mechanisms targeting complex eukaryotic symbionts. With this in mind, it seems likely that activation and use of these immune mechanisms, distinct from mechanisms directed toward symbiotic bacteria and yeast, is necessary for training and regulation of host immunity. A list of complex eukaryotic symbionts and their known health benefits in humans is shown in Table 1. Although more is known about the health benefits of helminths than of protists in humans,^112^^,^^118^data from animal studies support the view that protists have similar effects as helminths. For example, exposure to *Blastocystis* ST3 improves outcomes in experimentally induced colitis,^119^ and exposure to *Plasmodium berghei yoelii* prevents lupus and suppresses arthritis in laboratory animals.^32^^,^^33^ Nevertheless, as mentioned above, because the reproduction and transmission of many helminth species is readily controlled in the setting of high-income countries, these organisms are preferred over protists for ongoing efforts directed at controlled re-introduction of complex eukaryotic symbionts in high-income countries.

## Further evidence that the detrimental effects of all complex eukaryotic symbionts do not always outweigh potential benefits

There is absolutely no question that complex eukaryotic symbionts can, in particular cases, cause profoundly debilitating disease and even mortality. For that reason, biome reconstitution will never be considered with such pathogens as *A. lumbricoides*, *Strongyloides stercoralis* and *P. falciparum*, which cause substantial morbidity in humans. Indeed, the interaction between helminths and COVID-19 is not always helpful for patients. Our systematic literature search using the terms helminths and COVID-19 (see discussion above) revealed concerns emerging in 2020 of hyperinfection with *Strongyloides* following immunosuppressive treatment for COVID-19,^120^ with more than a dozen subsequent reports regarding the issue. Others have very reasonably raised concerns that debilitating infection with helminths will decrease the body’s ability to resist infection from SARS-CoV-2.

Wolday’s study^19^ showed no evidence that the presence of complex eukaryotic symbionts was causing harm. For example, abdominal pain and diarrhea, two potential signs of intestinal parasitism, were markedly less in individuals with complex eukaryotic symbionts: 7.5% and 6.9% of patients without complex eukaryotic symbionts had abdominal pain and diarrhea, respectively. In contrast, only 3.2% and 2.5% of patients with complex eukaryotic symbionts had abdominal pain and diarrhea (p = 0.014 and 0.009, respectively). On the other hand, potentially hidden effects of long term exposure to some complex eukaryotic symbionts might exist,^121^ and snap-shot surveys in time may not reflect the effects of long-term exposure. Nevertheless, if exposure to complex eukaryotic symbionts prevents mortality because of acute infection, then the cost/benefit ratio is almost certainly favorable regardless of the long-term effects.

Wolday noted that individuals with complex eukaryotic symbionts had fewer comorbid conditions, including diabetes and hypertension, than did individuals without such symbionts. Such findings might suggest some benefit to complex eukaryotic symbionts other than the predicted protection from severe COVID. Such benefits would not be surprising given the immunomodulatory role of complex eukaryotic symbionts.^51^^,^^53^^,^^54^^,^^55^ Furthermore, large, prospective studies^122^^,^^123^ demonstrate that, for the human population as a whole, detrimental effects of helminths at the population-level are apparently not significant when levels of colonization are low to moderate and potential long-term effects are not evaluated. Similarly, the presence of some protists is apparently benign in many cases.^112^^,^^124^ Thus, consistent with Wolday’s observations, all helminths and protists are not apparently parasitic. At the same time, Wolday’s observations do not in any way minimize the impact of particularly pathogenic helminths or protists, the impact of excessive exposure to such organisms that are benign at low to moderate levels, or the impact of such organisms on individuals with compromised immune systems. In this view, complex eukaryotic symbionts are identical to bacteria in that the nature of the symbiotic relationship, whether parasitic or mutualistic, is situational.^34^

## Some responses to SARS-CoV-2 are apparently not affected by biome alteration

Early in the COVID-19 pandemic, concerns were raised that helminth co-infection may suppress the immune response so that the human body cannot defend itself against SARS-CoV-2.^125^^,^^126^ However, helminths and protists are not generally immunosuppressive, per se, but rather are immunoregulatory.^112^^,^^127^ Thus, as might be expected, helminths and protists do not generally make individuals susceptible to common infections, and some aspects of immune function remain apparently unaltered by the presence of complex eukaryotic symbionts. Consistent with this view, although Wolday et al. observed an apparently profound impact of complex eukaryotic symbionts on the severity of COVID, as predicted, they found no such apparent effect on the loss of sense of taste or smell that is associated with COVID-19 (7.7% and 8.1% loss of taste/smell without and with complex eukaryotic symbionts respectively; p = 0.847). Loss of taste and smell, direct effects of viremia on the non-neuronal cells supporting taste bud function,^128^ can be taken as a surrogate marker for infection intensity in the mucosa of the upper GI tract. Thus, Wolday’s results^19^ suggest that immune interactions with complex eukaryotic symbionts do not play a major role in modulating viremia-associated morbidity during the initial stage of SARS-CoV-2 infection. In contrast, complex eukaryotic symbionts do modulate the progression of severe COVID, which is due in large part to immune system over-reaction. The critical observation here is that morbidity during the initial stage of infection *was not made worse* by the presence of complex eukaryotic symbionts. That is to say, no evidence was found suggesting that complex eukaryotic symbionts are immunosuppressive in the sense that they lead to increased viral load and failure to clear virus.

## Conclusions and future directions

The beneficial immunomodulatory effects of complex eukaryotic symbionts were first discovered before 1970.^29^^,^^30^^,^^31^^,^^32^^,^^33^ Unfortunately, the knowledge appears to have been all but lost in the field of immunology for decades. The hygiene hypothesis was proposed two decades later,^129^^,^^130^^,^^131^ but this hypothesis was focused more on childhood infections rather than lost symbionts. An emphasis on symbiotic helminths eventually retook center stage and displaced the hygiene hypothesis,^7^^,^^51^^,^^74^^,^^132^ giving rise to models described as “old friends” and “biota alteration”, but that shift in thinking took almost two more decades. Although it is now recognized that protists may afford some of the same immunoregulatory benefits as helminths, helminths are, as mentioned above, preferred for biota reconstitution over protists for practical reasons: The reproduction and distribution in the human population of helminths can be readily controlled, whereas the same does not hold true for protists.

**Figure 3:**
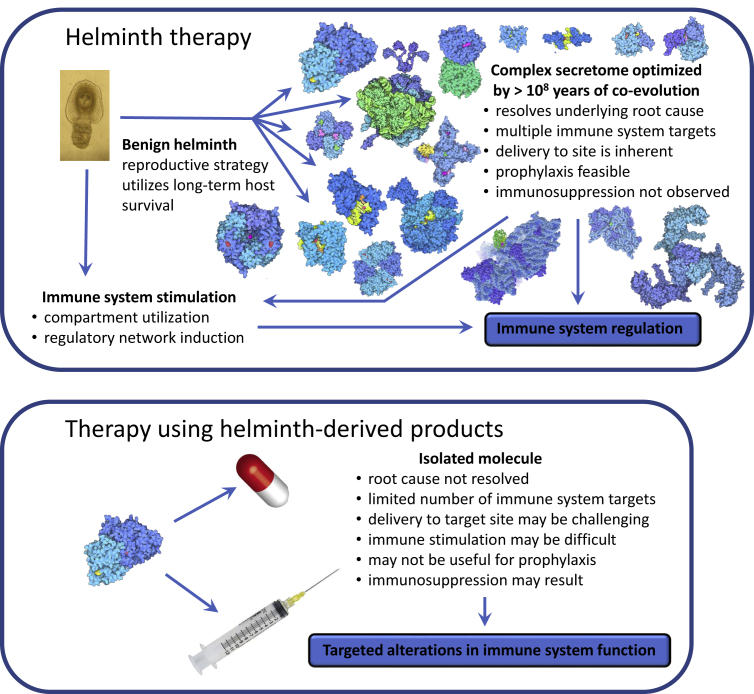
Helminth therapy versus therapy using helminth-derived products Helminth therapy offers several advantages over therapy using helminth-derived products. Although potential stigma associated with the use of live helminths in therapy is apparently not a significant hurdle,^77^ some financial hurdles may exist for the approach as it moves through regulatory channels.^113^^,^^114^^,^^133^ Illustrations of individual molecules in the diagram are taken from the protein data bank^134^ are shown for illustrative purposes only, and do not necessarily reflect molecules found within any helminth secretome. The cysticercoid life stage of the tapeworm *Hymenolepis diminuta* is shown as a representative, benign helminth.^19^

As the field of immunology struggled with its understanding of the origins of pandemics of chronic inflammatory disease, the ability of helminths to suppress immune function was, ironically, widely appreciated.^4^^,^^5^ With this knowledge, numerous labs attempted to develop helminth derived products into anti-inflammatory drugs. Unfortunately, such efforts are based on a pharmaceutical-based perspective that does not take into account the complexity of symbiotic biological relationships. Because symbiotic relationships cannot be recapitulated by single molecules, we have predicted that it will be impossible to develop helminth derived products that effectively eliminate chronic immune disorders.^35^ As shown in Figure 3, benign helminths offer substantial advantages over helminth derived products that may be difficult if not impossible to overcome. The bottom line is that, despite immeasurable suffering from chronic inflammatory conditions in high-income countries, and despite discovery of the role of complex eukaryotic symbionts in those conditions more than half a century ago, reconstitution of the human biome has not progressed past infancy. The fact that clinical mortality resulting from the COVID-19 pandemic was predictably^3^ made worse by the loss of complex eukaryotic symbionts highlights the tragedy of this lack of progress.

If Wolday’s study^19^ is any indication, preemptive biome reconstitution could have alleviated the majority of the clinical impact of COVID-19, which in turn may have alleviated much of the social and economic impact of the pandemic. This situation points toward the vital importance of translating fundamental observations into application. Indeed, simple observations that milkmaids do not acquire severe smallpox and that bacteria cannot grow near some colonies of mold were translated into vaccines and antibiotics, saving untold number of lives. These discoveries were made before the differences between T-cells and B-cells was established. That is to say, translating basic observations into life-saving technology does not require a detailed comprehension of immune function. Rather, the motivation to perform the translation is the only requirement.

Several factors should be weighed when considering how Wolday’s study might inform us regarding the potential impact of complex eukaryotic symbionts on individuals in high income countries. One issue, mentioned above, is that Wolday compared groups with and without active colonization of the intestine, and their group without active colonization may still have a considerable amount of exposure to complex eukaryotic symbionts. Another issue is the fact that some individuals, even in the most affluent countries, still have complex eukaryotic symbionts. For examples, the prevalence of the protist *Blastocystis*^135^^,^^136^^,^^137^ and of the helminth *Enterobius vermicularis*^138^ is still significant in some high-income countries, and some impoverished areas within high income countries may have exposure to helminths comparable to that of low-income countries.^139^ In addition, the average age and health of individuals in high-income countries is substantially different than that of the individuals who participated in Wolday’s study. With these considerations in mind, it is evident that the only way to know with certainty the impact of reintroducing complex eukaryotic symbionts on the immune responsiveness of individuals in high income countries is to directly measure the effects of biome reconstitution on individuals in those countries. However, given that epigenic effects can be generational, it may take decades or even a century before the full beneficial effects of biome reconstitution can be realized in high-income countries.
